# A numerical model of birch pollen emission and dispersion in the atmosphere. Model evaluation and sensitivity analysis

**DOI:** 10.1007/s00484-012-0539-5

**Published:** 2012-03-22

**Authors:** Pilvi Siljamo, Mikhail Sofiev, Elena Filatova, Łukasz Grewling, Siegfried Jäger, Ekaterina Khoreva, Tapio Linkosalo, Sara Ortega Jimenez, Hanna Ranta, Auli Rantio-Lehtimäki, Anton Svetlov, Laura Veriankaite, Ekaterina Yakovleva, Jaakko Kukkonen

**Affiliations:** 1 Finnish Meteorological Institute, Helsinki, Finland; 2 Main Geophysical Observatory, Petersburg, Russia; 3 Adam Mickiewicz University, Poznań, Poland; 4 Medical University Vienna, Vienna, Austria; 5 Russian State Hydrometeorological University, Saint Petersburg, Russia; 6 Finnish Forest Research Institute, Vantaa, Finland; 7 Universitat Autonoma de Barcelona, Barcelona, Spain; 8 EVIRA, Helsinki, Finland; 9 University of Turku, Turku, Finland; 10 Institute of the Industrial Ecology Problems of the Nort Kola Science Center, RAS, Apatity, Russia; 11 University of Siauliai, Siauliai, Lithuania

**Keywords:** Pollen dispersion, Dispersion model evaluation, Quality of pollen forecast

## Abstract

An evaluation of performance of the System for Integrated modeLling of Atmospheric coMposition (SILAM) in application to birch pollen dispersion is presented. The system is described in a companion paper whereas the current study evaluates the model sensitivity to details of the pollen emission module parameterisation and to the meteorological input data. The most important parameters are highlighted. The reference year considered for the analysis is 2006. It is shown that the model is capable of predicting about two-thirds of allergenic alerts, with the odds ratio exceeding 12 for the best setup. Several other statistics corroborate with these estimations. Low-pollen concentration days are also predicted correctly in more than two-thirds of cases. The model experiences certain difficulties only with intermediate pollen concentrations. It is demonstrated that the most important input parameter is the near-surface temperature, the bias of which can easily jeopardise the results. The model sensitivity to random fluctuations of temperature is much lower. Other parameters important at various stages of pollen development, release, and dispersion are precipitation and ambient humidity, as well as wind direction.

## Introduction

Long-range transport of pollen released from natural vegetation has been known for decades (Erdtman 1937). It has been shown repeatedly that only a small fraction of emitted pollen grains can travel far from the source, but the amount of pollen released by widespread wind-pollinated species, such as birch, is so large that the concentrations can still amount to hundreds of pollen grains per cubic metre even thousands of kilometres away from the source (D’Amato et al. 2007; Siljamo et al. 2008b; Skjøth et al. 2007, 2009; Sofiev et al. 2012a). This can pose an allergic threat for sensitive individuals and, being transported to other climatic zones, cause allergy outbreaks also outside the local flowering season of these regions (Viander and Koivikko 1978).

The episodic character of long-range pollen transport events makes them difficult to predict even using sophisticated tools such as modern atmospheric dispersion models (Efstathiou et al. 2011; Sofiev et al. 2006a). More common regional-scale dispersion of pollen also poses challenges for such models, but of different character: pollen release during the main flowering season depends on many dynamic parameters, such as air temperature, humidity, wind velocity, and precipitation rate (e.g. Helbig et al. 2004; Mullins and Emberlin 1997; Rempe 1938; Vogel et al. 2008; see also the companion paper by Sofiev et al. 2012b), whose impact is strong at regional scales. Finally, flowering season timing can vary strongly even within small regions (Siljamo et al. 2008b).

With significant uncertainties involved at every step of pollen forecasting, model evaluation and analysis of its sensitivity to input parameters and simulation setups have become a crucial part of the system operational cycle.

Evaluation of chemistry–transport models is a comparatively well-known area and a large number of various criteria and statistical measures have been suggested for the task (Schlünzen and Sokhi 2009). However, for each type of model application, a limited number of statistical measures is usually selected in direct connection with the purpose of the evaluation (Sofiev 1999). One possible classification of model applications, and the corresponding evaluation methodology, was developed within the AQMEII initiative (http://aqmeii.jrc.ec.europa.eu) (Dennis et al. 2009). The AQMEII initiative distinguished diagnostic, operational, probabilistic, and dynamic types of model evaluation and suggested the most typical statistical measures and procedures for each of them.

Another initiative, FAIRMODE, is aimed at developing a standardised methodology for the pre-operational evaluation of the models that are going to be used for the atmospheric composition forecast and re-analysis (http://fairmode.ew.eea.europa.eu), (Denby 2011).

The goal of the current paper is to evaluate the System for Integrated modeLling of Atmospheric coMposition (SILAM) in application to birch pollen dispersion in Europe, which is described in a companion paper (Sofiev et al. 2012b). Details of the SILAM system can be also found in Sofiev et al. (2006b, 2008). The spring of 2006 is taken as the reference period as it includes both regional and, one of the ever-strongest episodes of, long-range transport of birch pollen during the last decade.

The second goal of the paper is to study the sensitivity of the system to meteorological input and setup of the emission module. In particular, differences in model behaviour with the ECMWF and HIRLAM meteorological input and their dependence on the internal system setup are studied.

## Input data and methodology of the evaluation

### Pollen observations and phenological data

Regular birch pollen observations are available from the European Aeroallergen Network (EAN, https://ean.polleninfo.eu/Ean), which receives data from about 35 countries and about 300 sites. Pollen observations began in 1974 but the bulk of the data was collected after 1985.

In the current study, pollen observations were used for two different purposes:

1. Twenty years of pollen data (1980–2000) were combined with the phenological data for determination of the first flowering dates, which were further used for the heat sum threshold calculations following the procedure of Sofiev et al. (2012b);
2. the 2006 dataset was used for verification of the SILAM dispersion results, i.e. for direct comparison of observed and predicted pollen concentrations in air.

For evaluating the first flowering date, the following completeness requirements were applied:

- the station must report the data for at least 45 days during April–May (general completeness threshold of 75  %)
- the number of reported days in April should not be lower than that in March (completeness homogeneity requirement)
- using the SILAM simulations for indication of presence of pollen in the air, it was required that:
  - the station reported the observations for at least 80  % of days when the non-zero pollen concentrations were predicted by the model (active pollen season completeness)
  - since the SILAM simulations with different model setup differ from each other, the above active pollen season completeness requirement appeared ambiguous and, if all runs are taken into account, very tight. Therefore, the station was also accepted if, for any SILAM simulation, it missed not more than 1 day with non-zero predicted concentrations (relaxation of the active pollen season completeness).

Application of the above requirements resulted in a reduction of the number of included stations by about 25  %. For instance, for 2006, a total of 155 stations were included in the evaluation of the start and end of flowering out of 213 stored in the archive.

The task of the model evaluation evidently did not require any station filtration; we simply used only those SILAM predictions for which the corresponding observations existed.

Phenological data for the temperature sum threshold computations were taken from a database collected within the POLLEN project of the Academy of Finland (Siljamo et al. 2008a). The database contains information on bud burst, leaf unfolding, and the first flowering date from 15 countries for more than 6,500 stations. Most of the data were collected after 1985 but the oldest observations go back to the middle of the eighteenth century. The longest continuous time series covers a time period of over 30 years. Overall, the database contains about 60,000 individual data entries. The bulk of the information is on the dates of leaf unfolding. Since birch pollination usually starts a couple of days earlier than that, leaf unfolding dates were used as a substitute for the first flowering day.

### Setup of SILAM simulations

The computational domain of all model simulations covered the whole of Europe and used the birch forest distribution of Sofiev et al. (2006a) with a split to nine sub-regions evaluated separately (Fig. 2). The simulations started from 1 March 2006 and covered a period of 4 months. The runs were repeated four times (Table 1): for two heat sum threshold maps (based on phenological and aerobiological observations—see the Results section for details) and for two sources of the meteorological data: HIRLAM (high resolution limited area model (Unden et al. 2002)) and ECMWF (European Centre for medium range weather forecast, http://www.ecmwf.int). The horizontal resolution of the HIRLAM model was 0.2°; the model had 40 vertical hybrid levels. The ECMWF model horizontal resolution was 0.25°; the model had 91 vertical hybrid levels. The SILAM computational resolution in all cases was 0.25°. The meteorological fields were supplied to SILAM every 3 h in all cases. We used the shortest possible forecasts but excluded the analysis fields for technical reasons. Thus, the +3 h and +6 h forecasts were taken in case of HIRLAM and from +3 h till +12 h data were taken in case of ECMWF.

**Table 1:** Setups of the system for integrated modeling of atmospheric composition (SILAM) pollen forecasting system. *Hirlam* High resolution limited area model, *ECMWF* European Centre for medium range weather forecast, *LU* leaf unfolding, *COMB* combined leaf unfolding and pollen counts

Setup name	NWP model (horizontal resolution, no. of vertical levels, length of forecasts)	Phenological model	Correction factor for SILAM pollen concentrations
Hirlam LU	HIRLAM (0.2º, 40, +3 h and +6 h)	Leaf unfolding-based temperature sum threshold	2.33
Hirlam COMB	HIRLAM (0.2º, 40, +3 h and +6 h)	Combined leaf unfolding and pollen counts based temperature sum threshold	2.39
ECMWF LU	ECMWF (0.25º, 74, +3 h, +6 h, +9 h, +12 h)	Leaf unfolding based temperature sum threshold	3.03
ECMWF COMB	ECMWF (0.25º, 74, +3 h, +6 h, +9 h, +12 h)	Combined leaf unfolding and pollen counts based temperature sum threshold	2.94

### Evaluation procedure

The main application area of the SILAM pollen computations is related to predictions of high pollen concentrations that lead to outbreaks of allergy. The model is also used increasingly for the longer-term assessment of the pollen dispersion. Therefore, we aimed at evaluating (1) the overall agreement with observations, and (2) the model ability to predict exceedances of a certain pollen concentration threshold. Since threshold-based statistics have low stability with regard to model and observational uncertainties (Sofiev and Tuovinen 2001), it was important to collect sufficient data to allow for large-volume averaging. Therefore, we concentrated on analysis of the whole pollen season for the large regions in Fig. 2, leaving out considerations of specific episodes and individual time series.

Two sets of thresholds were used for the above evaluation tasks. For studying the ability of the general model to reproduce the concentration distribution, we used five classes after Rantio-Lehtimäki et al. (1991): zero (less than 1 pollen grain m^−3^), low (1–10 pollen grains m^−3^), moderate (10–100 pollen grains m^−3^), high (100–1,000 pollen grains m^−3^) and very high (>1,000 pollen grains m^−3^). This classification covers the whole range of birch pollen concentrations and allows for analysis of a concentration histogram with a sufficient number of cases falling into each range, so that the statistical computations have sufficient precision.

For evaluating the quality of the threshold exceedance predictions, we needed only one threshold. Its value was selected to be in the middle of the central bin of the classification of Viander and Koivikko (1978): 50 pollen grains m^−3^, which is also in the centre of the above classification. Out of the total number of observations *N*_total_ (all daily values from all stations), *N*_O_low_ and *N*_O_high_ were defined as the number of daily observed concentrations below and above 50 pollen grains m^−3^, respectively. For the same days and locations, *N*_M_low_ and *N*_M_high_ were defined as the number of days with the model predicted concentration below and above 50 pollen grains m^−3^, respectively. Evidently, ${N_{{O\_low}}} + {N_{{O\_high}}} = {N_{{M\_low}}} + {N_{{M\_high}}} = {N_{{total}}}$. Then, *N*_M_low_O_low_, *N*_M_low_O_high_, *N*_M_high_O_low_, *N*_M_high_O_high_ represent all combinations of the relation between the model predictions and observations, ${N_{{M\_low\_O\_low}}} + {N_{{M\_high\_O\_low}}} + {N_{{M\_low\_M\_high}}} + {N_{{M\_high\_O\_high}}} = {N_{{total}}}$.

With these notations, the fraction of correct predictions (model accuracy, MA) is:

1 $$ MA = \frac{{{N_{{M\_low\_O\_low}}} + {N_{{M\_high\_O\_high}}}}}{{{N_{{total}}}}} $$

The Hit Rate, HR (also called probability of detection, POD) is the fraction of correct “high” predictions:

2 $$ POD,HR = \frac{{{N_{{M\_high\_O\_high}}}}}{{{N_{{M\_high\_O\_low}}} + {N_{{M\_high\_O\_high}}}}} $$

The false alarm ratio (FAR) is the fraction of incorrect “high” predictions:

3 $$ FAR = \frac{{{N_{{M\_high\_O\_low}}}}}{{{N_{{M\_high\_O\_low}}} + {N_{{M\_high\_O\_high}}}}} $$

Probability of false detection (POFD) shows the fraction of low-concentration days predicted as “high”:

4 $$ POFD = \frac{{{N_{{M\_high\_O\_low}}}}}{{{N_{{M\_high\_O\_low}}} + {N_{{M\_low\_O\_low}}}}} $$

Finally, the odds ratio (OR) shows how much higher are the chances to get a “high” day than a “low” day if the model prediction is “high”:

5 $$ OR = \frac{{POD}}{{POFD}} $$

The meaning of OR is similar to that of the difference POD−POFD, also known as the Hansen-Kuiper or True Skill Score.

## Results

### Temperature sum thresholds for the first flowering date

A specific problem that has to be addressed with regard to the start of flowering is the suitability of the phenological and pollen data for its estimation. This date is available directly from the phenological records but the uncertainty in many regions is large (Siljamo et al. 2008a). We have also computed start of flowering from EAN data following the 2.5   % criterion after Goldberg et al. (1988) and Veriankaitė et al. (2010): the flowering season was declared started as soon as the cumulative pollen count reached 2.5  % of the annual sum. Similarly, the end of the flowering season was computed with the 95  % criterion. For Northern Europe, 2.5  % appeared to be too low due to significant impact of pollen long-range transport at the beginning of the pollen season (Ranta et al. 2006). Therefore, we also used a 5  % criterion for the flowering starting date for more effective filtration of the long-range transport episodes at the beginning of spring. Throughout the paper, the criterion is stated each time the threshold value is applied.

**Fig. 1:**
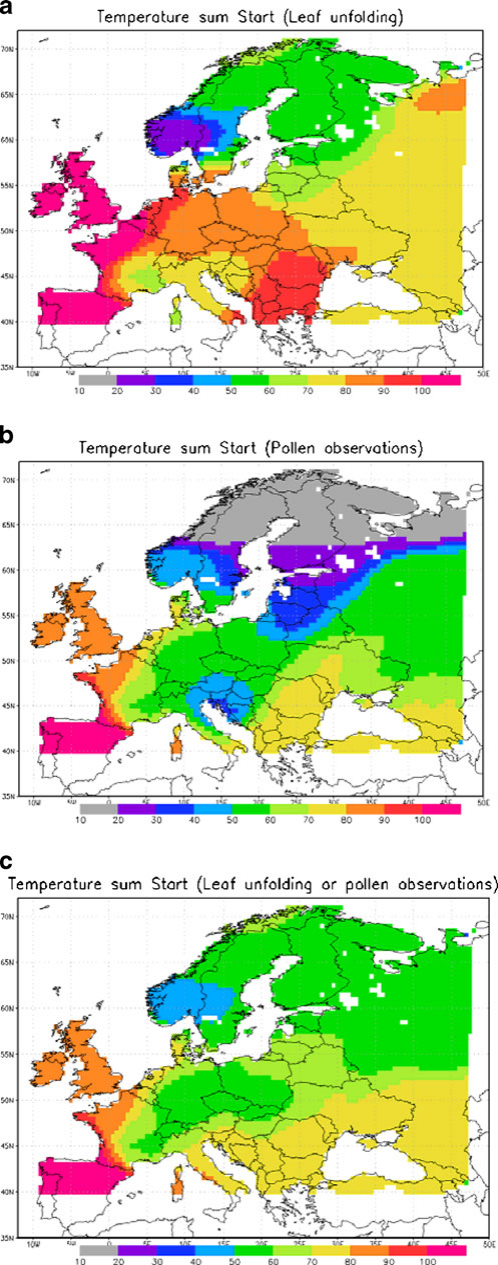
**a**–**c** Temperature sum threshold maps (degree days) used in system for integrated modeling of atmospheric composition (SILAM) birch pollen simulations. Start of accumulation is 1 March, cut-off temperature is 3.5 °C. **a** Using leaf unfolding phenological observations, **b** using pollen counts, **c** using combination of pollen counts and leaf unfolding

To compare model sensitivity to the threshold computation algorithm, we generated three threshold maps for the temperature sum (Fig. 1). The first map is the same as in the companion paper (Sofiev et al. 2012b): it is based solely on leaf unfolding data (hereafter called LU map). The second map is based solely on pollen counts and 2.5  % criterion (OBS map). The third map is based on the combination of leaf unfolding data and pollen observations (hereafter COMB or combination map). For the combination map, pollen counts were used in Central, Sothern and Eastern Europe wherever available. In the Northern Europe they were replaced with the leaf unfolding phenological observations to exclude the influence of early-spring long-range transport.

Upon construction, it turned out that the OBS map manifested unrealistically low temperature sum thresholds in Northern Europe (Fig. 1) due to the impact of early events of long-range transport. It was therefore excluded from further consideration, thus leaving only the LU and COMB threshold maps for the analysis.

### Timing of flowering season

The propagation of the flowering season in 2006 for both meteorological input datasets and LU threshold map, as well as the difference between the HIRLAM and ECMWF temperature sums, are shown in Fig. 3 for 1 April, 15 April, 29 April and 13 May 2006.

On 1 April, birch flowering started in Southern Europe, and the difference between flowering areas predicted with HIRLAM and ECMWF meteorological inputs is small. The HIRLAM model forecasts lower temperatures in the north (latitude > 60°N) and over the sea areas (Fig. 3, rightmost column) but the difference does not (yet) result in disagreement of the predicted spread of the flowering season.

By the middle of April, warmer temperatures in the HIRLAM forecasts for Central Europe start affecting the flowering patterns, so that the HIRLAM-driven simulations predict the on-going season over substantially larger regions than the ECMWF-driven run. This tendency continues towards the end of April when flowering finishes over most of Central Europe according to the HIRLAM-based predictions but not according to ECMWF-based simulations.

In May, the impact of HIRLAM-predicted low temperatures in northern areas and over water result in the differences between runs being largely evened out, so that the predicted flowering areas are similar to each other. Actually, the flowering season is predicted to be finished over a even larger area in the ECMWF-driven run than in the HIRLAM-based one.

**Fig. 2:**
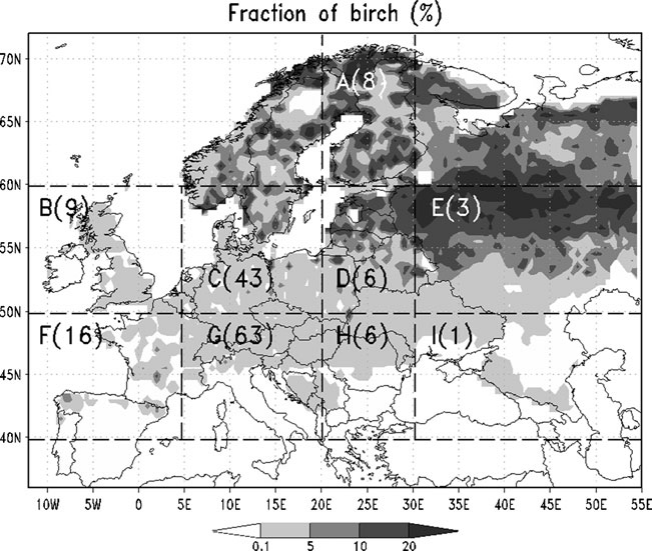
Fraction of birch (adapted from Sofiev et al. 2006a) used in the simulations. Considered sub-regions are delineated and labelled. The numbers of accepted stations for each sub-region is shown in *brackets*

**Fig. 3:**
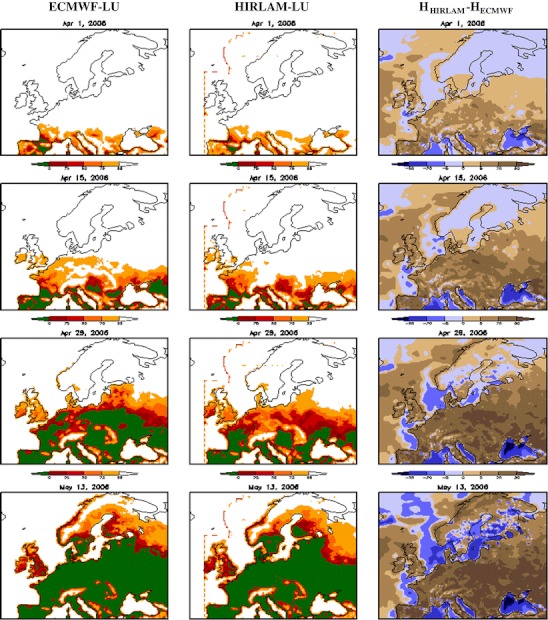
Season propagation in 2006: 1 April, 15 April, 29 April and 13 May. *Left panels* Pollen still in catkins ( %) in high resolution limited area model (HIRLAM) leaf unfurling (LU) setup; *middle panels* pollen still in catkins ( %) in ECMWF (European Centre for medium range weather forecast) LU setup; *right panels* cumulative temperature sum difference HIRLAM−ECMWF, [K]. Colours for pollen: *white* no flowering yet, *yellow/orange* on-going flowering (first half of season), *red/brown* on-going flowering (second half), *green* flowering over. Colours for temperature sum: *brown* Hirlam is warmer; *blue* ECMWF is warmer

For comparison with EAN observations, flowering start dates were estimated from modelled and observed pollen concentrations following the 5  % criterion. The results of comparison for the regions outlined in Fig. 2 are presented in Table 2. Among the four model setups, the run based on the ECMWF meteorological data and the LU threshold map seems to provide the most accurate results. This setup showed just 1 day early bias of the start of flowering. The largest bias was demonstrated by the HIRLAM-COMB setup, which is more than 1 week too early. The reason is that the HIRLAM predictions of the 2 m temperature are practically always warmer than those of the ECMWF in Central and eventually even in Northern Europe (Fig. 3, right-hand-side column).

**Table 2:** Bias (observation-forecast) and root mean square error (RMSE) of the first flowering day prediction, computed from observed and predicted pollen counts using the 5  % criterion. Positive bias implicates too early and negative bias means too late a predicted date. Unit = [day]. Areas are as in Fig. 2

	SILAM setup							
Area (nbr of stations)	HIRLAM				ECMWF			
	LU		Combination		LU		Combination	
	BIAS	RMS	BIAS	RMS	BIAS	RMS	BIAS	RMS
All obs. (155)	4.4	6.3	7.6	9.1	1.0^a^	4.5^a^	4.2	6.3
A (8)	3.5	4.2	4.0	4.6	−0.4^a^	1.4^a^	−0.3	1.4
B (9)	−2.6	5.6	1.4^a^	7.3	−2.8	3.9	1.7	2.7^a^
C (43)	4.3	5.2	9.7	10.4	1.7^a^	2.7^a^	4.9	5.9
D (6)	2.7	3.2	5.7	7.1	−0.5^a^	1.8^a^	1.2	2.6
E (3)	2.0	5.0	5.3	6.1	−3.0	4.2^a^	0.0^a^	4.2
F (16)	6.6	8.6	8.6	9.9	2.8^a^	6.8^a^	6.5	8.2
G (63)	5.6	7.1	8.0	9.2	1.5^a^	5.2^a^	4.7	7.2
H (6)	0.5^a^	3.7	3.3	4.7	−1.7	3.5^a^	2.8	4.2
I (1)	1.0^a^	1.0^a^	1.0^a^	1.0^a^	−4.0	4.0	−4.0	4.0

^a^Smallest RMSE and bias for each region

In general, the SILAM performance with the ECMWF meteorological driver is better than with the HIRLAM input—for both LU and COMB temperature sum threshold maps (Table 2). Similarly, the LU threshold map allows for better scores than the COMB one. The only exception is the UK where birch starts pollination very early.

Region-wise, the prediction of the first flowering day by the ECMWF-LU setup had the smallest bias and RMSE in Finland (area A, ∼0.5 day too late, RMSE < 2 days) and in the Baltic States (area D). The most challenging areas appeared to be in marine climate (areas B and F), where the model was either almost 3 days late (UK) or 3 days early as in France and Spain. RMSE was biggest in France and Spain (almost 1 week), and in the mountains (area G, about 5 days).

Predicting the end of flowering, which was estimated with 95  % criterion from the observed and modelled pollen concentrations, appeared to be challenging (Table 3). The model tends to predict too long a flowering season. Consequently, the HIRLAM-COMB setup, which predicts start of flowering ∼1 week too early, performs best for the end of the flowering season (only 3 days too late). However, if flowering season durations are compared, the prediction is more than 10 days too long. The ECMWF-LU setup does not give the best prediction of the end of the flowering season but has twice as small an error as that of HIRLAM-based runs when the length of the flowering season is considered (5.7 days too long).

**Table 3:** Bias (observation−forecast) and RMSE of the last flowering day prediction, computed from observed and predicted pollen counts using the 95  % criterion. Positive bias implicates too early and negative bias means too late predicted date. Unit = [day]. Areas as in Fig. 2

	SILAM setup							
Area (nbr of stations)	HIRLAM				ECMWF			
	LU		Combination		LU		Combination	
	BIAS	RMS	BIAS	RMS	BIAS	RMS	BIAS	RMS
All obs. (155)	−3.5	6.8	−3.1^a^	6.6^a^	−4.7	7.1	−3.9	7.5
A (8)	−3.9	7.0^a^	−4.0	7.1	−2.1^a^	7.1	−2.1^a^	7.1
B (9)	−3.2^a^	4.3^a^	−3.2^a^	4.3^a^	−7.7	9.7	−5.3	7.7
C (43)	−1.0	2.8^a^	−1.0^a^	2.8	−1.6	3.4	−1.3	3.4
D (6)	0.3^a^	1.4^a^	0.5	1.6	−0.8	2.0	−0.3^a^	1.5
E (3)	−1.7	8.2	0.7^a^	5.4^a^	−11.3	11.4	−6.0	7.9
F (16)	−3.4	5.3	−2.4^a^	4.7^a^	−3.6	6.9	−2.6	6.5
G (63)	−6.2	9.6	−5.8^a^	9.3^a^	−7.6	10.7	−6.6	10.1
H (6)	1.0	1.8^a^	1.4	2.0	−1.0	2.3	−0.4^a^	1.9
I (1)	0.0^a^	0.0^a^	1.0	1.0	0.0^a^	0.0^a^	0.0^a^	0.0^a^

^a^Smallest RMSE and bias for each region

Region-wise, the prediction of the end of the flowering season is most accurate for the Baltic States (area D) and worst in the mountainous areas of Central Europe (area G).

### Pollen concentrations in the air

Before evaluating the absolute pollen concentrations in the air, the model output was renormalised to compensate for the missing mechanism for the inter-annual variability of the pollen production. For each of the four SILAM runs, the seasonal total for all EAN stations was scaled to be equal to the mean cumulative count observed by these stations until 31 May. Correction factors for these runs were slightly different (varying between 2.3 and 3) but sufficiently close to each other to suggest comparable patterns of pollen distribution (Table 1).

#### Representation of concentration distribution; selection of best setup

**Fig. 4:**
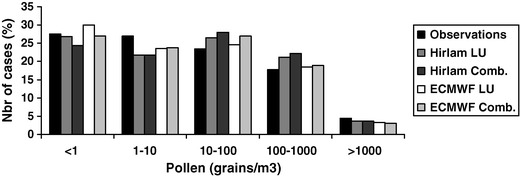
Number of cases (percent of 11,210, excluding the no observation cases) for zero (<1 grain m^−3^), low (1–10/m^−3^), moderate (10–100/m^−3^), high (100–1,000/m^−3^), and very high (>1,000/m^−3^) pollen concentrations

The distribution of concentrations over the above-defined five concentration ranges (Fig. 4) are within ∼5  % of the observed distribution—for all four SILAM setups. Slight deviations, e.g. over-representation of zero cases by the ECMWF-LU run or general under-representation of low cases (1–10 pollen grains m^−3^) in favour of the higher rank by all four setups are within the uncertainty limits of the observations themselves (Burge 1992; Levetin et al. 2000; Solomon 1984). Therefore, one can conclude that the distribution of pollen concentrations is reproduced well by all model setups.

**Fig. 5:**
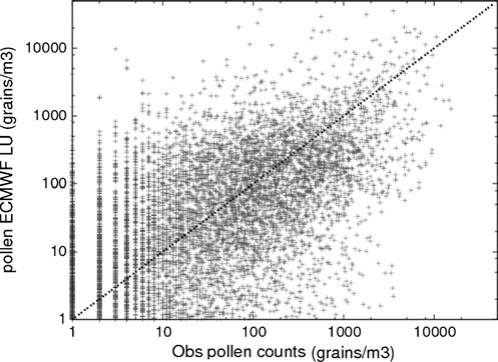
Observed daily pollen concentrations vs predicted daily pollen concentrations with the SILAM model using the ECMWF LU setup

The scatter plot of the observed and predicted concentrations (Fig. 5) shows quite a substantial scatter, with the model−measurement differences reaching up to an order of magnitude. However, the bulk of the predictions are within a factor of 3 to 4 from the observations.

**Fig. 6:**
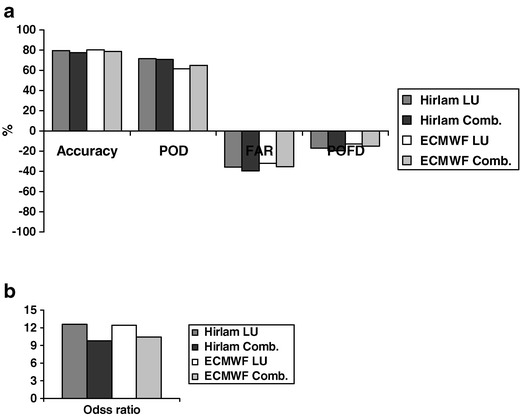
Statistical scores (1)–(5) of performance of the SILAM setups for spring 2006

For quantitative evaluation, the statistics Eqs. (1)–(5) were calculated with regard to the threshold of 50 pollen grains m^−3^ (Fig. 6). As one can see, the accuracy of the configurations was again quite similar. The difference between the highest scores (80  %) of the ECMWF-LU setup and the worst scores of HIRLAM-COMB setup (77  %) is not statistically significant.

The HR, however, showed some weak preference for HIRLAM-based setups (>70  %) over ECMWF-driven runs (<∼65  %). The “negative” scores related to false alarms (FAR and POFD) suggested that the ECMWF-based setups were marginally better than the HIRLAM ones. However, the LU threshold map was clearly a better choice than the combination map. This message is confirmed by the OR, which showed that: (1) the best odds of the “high” forecast being correct is more than 12 times higher than it being wrong; (2) the LU-based setups are clearly better than those based on the COMB maps. The impact of the meteorological driver was small.

Summarising, the ECMWF-LU configuration was slightly better than the others for all scores except for the hit rate, the second was the HIRLAM-LU setup, then the ECMWF-COMB, and the worst was the HIRLAM-COMB configuration. The difference between them is not large, so it is also possible to conclude that the system is quite robust with regard to features of the meteorological drivers and, to a less extent, the parameterisation of the heat sum threshold.

#### ECMWF-LU setup: probability of “good” prediction

For the extra analysis of the best setup, the five histogram ranges of Fig. 4 were grouped into three classes: zero or low (0–10 pollen grains m^−3^), moderate (10–100 pollen grains m^−3^), and high or very high (>100 pollen grains m^−3^). For observations, a fourth class “no-data” was introduced. The ECMWF-LU model-measurement pairs were distributed over these classes and the probabilities for the model prediction to hit the correct class were calculated (Table 4).

**Table 4:** Hit rates of the SILAM pollen predictions with the ECMWF weather data and LU threshold map

Observations	No data	0–10/m^−3^	10–100/m^−3^	100–1,000/m^−3^
ECMWF LU				
0–10/m^−3^	17 %	65 %	13 %	4 %
10–100/m^−3^	11 %	34 %	33 %	22 %
100–1,000/m^−3^	8 %	11 %	25 %	56 %

As seen from the first line of Table 4, the SILAM zero or low concentration predictions appeared correct in 65  % of cases (plus, most probably, the bulk of the 17  % of cases with no observations). Only 4  % of the “low” predictions were made for actually “high” days. The high predictions were correct in >56  % cases (plus a fraction of 8  % of no-data) and in only 11  % of cases were the actual values low.

Moderate concentrations were the most difficult for the current model: only about one-third of predictions fell in the correct class (plus a fraction of 11  % no-data cases). In fact, when the model predicted “moderate” class, the chances of observations being “low” or “high” were nearly the same as being “moderate” (34  %, 22  %, 33  %, respectively). This outcome is unusual for air quality forecasting and is discussed further in the next section.

A comparison of the best setup with those using the different threshold map (Table 5a) and meteorological driver (Table 5b) show that they do not really disagree with each other but rather tend to deviate within 10–20  % at most. The more significant variations are due to changes in the meteorological driver. Also, predictions of moderate concentrations were the most sensitive: only 58  % of cases predicted as “moderate” by the ECMWF-LU run fall into the same class if the NWP input is changed to HIRLAM (Table 5b). This corroborates well with the sensitivity analysis in our companion paper (Sofiev et al. 2012b), which shows the strong impact of meteorological parameters on pollen release.

**Table 5:** Distribution of predicted pollen concentrations using ECMWF data and LU threshold map ,and setups ECMWF input with COMB threshold map, or HIRLAM input with LU threshold map

ECMWF comb	0–10/m^−3^	10–100/m^−3^	100–1,000/m^−3^
ECMWF LU			
0–10/m^−3^	93 %	7 %	0 %
10–100/m^−3^	5 %	89 %	6 %
100–1,000/m^−3^	0 %	6 %	94 %
HIRLAM LU	0–10/m^−3^	10–100/m^−3^	100–1,000/m^−3^
ECMWF LU			
0–10/m^−3^	82 %	7 %	4 %
10–100/m^−3^	20 %	58	21 %
100–1,000/m^−3^	1 %	15 %	83 %

## Discussion

The evaluated birch pollen forecasting system is a combination of many sub-models, all of which have their strong and weak points. In particular, it uses meteorological fields from external NWP models and thus depends greatly on the quality of the latter. This dependence has clearly identifiable long- and short-term components.

### Estimation of flowering season start: uncertainties that propagate through long-term averaging

Prediction of flowering start is based on the long-term temperature sum computation, which is sensitive to the temperature bias in the NWP model. The accumulation period before the temperature sum threshold is reached can be as long as 2–3 months, and already a temperature bias of 0.5°C would lead to an error of 30–50 degree-days of the temperature sum. This level is comparable with the threshold itself (Fig. 1) and evidently leads to a prediction error of a week or more in the flowering start date. On the contrary, the zero-mean temperature fluctuations are averaged out very efficiently during this long accumulation time. The only exception is when these fluctuations happen near the cut-off temperature threshold (Sofiev and Tuovinen 2001) but this does not seem to be a frequent problem.

High sensitivity to biases is a general feature of all time-integrating algorithms and the only way to detect and correct such eventual temperature bias is the assimilation of observational phenological or aerobiological data. However, standard data assimilation approaches do not help with emission corrections. Therefore, sophisticated methods based on variational assimilation or ensemble filtering would be necessary to improve the stability of predictions (Elbern et al. 2007; Vira and Sofiev 2011).

### Uncertainties in the birch forest map and seasonal pollen production

Some static input data, such as the fraction of birch forest in the area and the total amount of pollen released during the whole spring, affect model accuracy directly and inherit their own uncertainties. There is still a strong lack of knowledge of the distribution of birch trees in Europe. An even more uncertain parameter is the total amount of pollen grains released from a unit area of birch forest during the specific flowering season. The inter-annual variation of flowering intensity can be predicted, to some extent, from the previous-year information but the procedure explains only a fraction of the variability and seems to be highly data-sensitive (Ranta et al. 2008).

Uncertainties in these static parameters propagate linearly to errors of the concentrations. In the present study, we normalised the annual pollen count to the mean observed values over Europe in 2006, thus correcting the bulk impact of these factors. However, regional inhomogeneities were not corrected, and contributed to the scatter in Fig. 5.

### Uncertainties in concentration predictions during the main season

In comparison with the pre-season, the estimation of pollen release and dispersion during the main flowering period poses challenges that are more familiar in air quality modelling practice. Precipitation and humidity can delay or inhibit flowering, wind and turbulence promote it, whereas transport patterns are affected by regional- and synoptic-scale weather, turbulence and other parameters describing the conditions of dispersion within and above the atmospheric boundary layer. Uncertainties in these data lead to scatter of the predicted concentrations with regard to observed values but their impact is usually short-term (a few hours or days). These sources of errors in pollen predictions are common also for other air quality characteristics—see, e.g. (Rouil et al. 2009).

Consideration of the 2006 time series (not shown), as well as practical experience from several years of system exploitation, showed that the accuracy of pollen concentration forecasts tends to be best for long-range episodes, which are usually caused by pollination in large, albeit remote, birch forests. This is because the small-scale irregularities of dense wide plumes are smoothed out during transport, thus improving the agreement with observations. Near the source, even a limited inaccuracy of the temperature sum prediction, birch tree distribution, or threshold values immediately affect agreement with nearby stations.

Experience with modelling airborne chemicals usually shows that air quality (AQ) models predict moderate concentrations well but experience difficulties with low and high levels (van Loon et al. 2007; Vautard et al. 2009). The pollen model behaviour appeared to be the opposite: peaks and lows were predicted better than moderate values (Table 4).

The behaviour of “standard” AQ models has clear explanations. Firstly, the highest atmospheric concentrations occur commonly under meteorological conditions that are difficult to simulate accurately (for instance, extremely stable atmospheric stratification, low-level temperature inversions, atmospheric re-circulation, etc.) (Kukkonen et al. 2005). The averaging over the model grid cell also smooths out the pattern and thus lowers the predicted peak concentrations. Secondly, in low concentration cases, numerous slow and local phenomena, being small and poorly reproduced, become significant due to suppression of the main mechanisms.

For the pollen model, there are several factors contributing to its peculiarity. One explanation is the specifics of birch distribution in Europe. In the north, vast forest areas with a substantial birch fraction constitute a very strong and wide-area source of pollen. In Central Europe, birch trees are still rather common but do not form forests, being spread over small pieces of wild land and urbanised areas. In the south, birch is an exotic tree. Over areas with a high fraction of birch forests, airborne pollen concentrations increase fast from low to high when pollination starts and quickly fade when the flowering season is over. These areas are quite well known and are captured in the map in Fig. 2. For such areas, dilution over the model grid cell is not a problem since the source of pollen also covers a wide area. This is the main reason why high pollen concentrations are reproduced quite well.

High scores for low concentrations simply mean that the flowering season is captured well by the emission module, so that low- or no-emission regions and time periods are correct.

Moderate concentrations are more typical of areas where birch is not a dominant tree species but is rather spread around and mixed with other vegetation, sporadically planted as an ornamental tree, etc. The quality of the birch map in such regions is expected to be lower, which affects concentration scores.

Pollen has another strong difference from chemical and most aerosol pollutants: it deposits quickly but even low concentrations and small fractions of the emitted amount reaching remote places still have a strong impact. Pollen counts can exceed 10,000 pollen grains m^−3^ in source regions but sensitive people can experience allergic symptoms if concentrations are as low as 10 pollen grains m^−3^, and a level of 100 pollen grains m^−3^ is considered abundant (e.g. D’Amato et al. 2007; Viander and Koivikko 1978). The importance of low pollen concentrations challenges the model, which should catch tails of several days-long episodes and also reproduce the start and end of local flowering when only a fraction of trees are pollinating.

### Comparison of model setups and input datasets

The results of this study indicate that ECMWF-driven setups perform better than those based on HIRLAM input, especially for prediction of the start of the flowering season. However, this does not necessarily mean that the ECMWF model predicts the temperature better. One has to keep in mind that the ERA-40 dataset (Uppala et al. 2005), which underlies temperature sum threshold maps, is also based on the ECMWF model (albeit a different version). To minimise this dependence on the NWP model, we used the analysed ERA-40 fields, which are obtained via data assimilation, but still the impact of the underlying model cannot be eliminated completely. Therefore, the better SILAM scores with ECMWF forcing should be considered as a confirmation of the overall system integrity rather than as a criterion for evaluation of input data quality. In this light, it is the stability of the system and its acceptable quality when using the HIRLAM data that should be considered as an important outcome of the inter-comparison.

## Conclusions

Evaluation of the SILAM birch pollen forecasting system has quantified the model capabilities to reproduce the observed distribution patterns and absolute levels of concentrations.

Both high and low concentration levels are reproduced correctly in about two-thirds of actual cases. The odds ratio required for the predicted high-concentration episode to be real exceeds 12 for the best model setup. Moderate concentrations are predicted with a lower confidence, which may indicate problems related to the birch forest map in regions where birch stands are scarce.

Region-wise, the best scores are demonstrated for Northern Europe, where birch forests are most abundant and information about their distribution is presumably the most accurate. Problematic regions appear to be in marine climates, where the impact of temperature and humidity may differ from that in the rest of the domain, and in mountainous terrain, where both NWP and SILAM resolutions were inadequate.

The importance of meteorological parameters varied in different parts of the flowering season. Before the flowering season, the long-term stability of temperature predictions was important for flowering start date. The error of this date varied from 1 to 2 days in the best-performing setup, to up to more than a week if the temperature prediction is biased in the NWP model.

Short-term processes influence pollen release and dispersion during the flowering season, adding up to the scatter between the model and the measurements.

As a result of the evaluation, the best-performing setup was selected for operational simulations: the ECMWF meteorological input and LU-based temperature sum threshold map. However, stability of the system with regard to variations in the input data allows use of any of the considered configurations without specific corrections in the system.
